# Investigation of Site-Specific Differences in Glycan Microheterogeneity by N-Glycopeptide Mapping of VEGFR-IgG Fusion Protein

**DOI:** 10.3390/molecules24213924

**Published:** 2019-10-30

**Authors:** Young Hye Hahm, Ju Yeon Lee, Yeong Hee Ahn

**Affiliations:** 1 Rophibio, Inc., Cheongju 28160, Korea; yhhahm@rophibio.com; 2 Korea Basic Science Institute, Biomedical Omics Research Group, Cheongju 28119, Korea; jylee@kbsi.re.kr; 3 Department of Biomedical Science, Cheongju University, Cheongju 28160, Korea

**Keywords:** biosimilar, fusion protein, site-specific glycan profiling, microheterogeneity, LC-ESI MS/MS

## Abstract

A biosimilar fusion protein VEGFR-IgG consisting of vascular endothelial growth factor receptors 1 and 2 (VEGFR-1, VEGFR-2) and the Fc portion of human IgG1 was prepared for this study. The prepared fusion protein was expected to possess a total of five N-linked glycosylation sites: two sites in the VEGFR-1 region, two sites in the VEGFR-2 region, and one site in the human IgG Fc region. For site-specific glycan analysis, the fusion protein was hydrolyzed with trypsin, and the resulting tryptic digests were analyzed by liquid chromatography–electrospray ionization tandem mass spectrometry (LC-ESI MS/MS). The expected N-linked glycosylation sites were successfully identified and site-specific glycopeptide mapping was completed by Integrated GlycoProteome Analyzer (I-GPA) for the resulting raw tandem mass data. Finally, it was clearly confirmed that N-linked glycans for each glycosylation site showed significantly different patterns in microheterogeneity, which may indicate certain functions for each glycosylation site in the protein. Based on the mapping results, the unique features in glycan microheterogeneity for the five glycosylation sites of VEGFR-IgG fusion protein were compared site-specifically and further discussed to understand the functional meaning of each glycosylation pattern.

## 1. Introduction

Worldwide development of biosimilar drugs has grown over the past decade in an attempt to decrease the burden of healthcare costs produced from costly biopharmaceutical drugs. Biosimilars are defined as biological medicinal products that are highly similar to and have no clinically meaningful differences from existing reference products. Many blockbuster IgG monoclonal antibodies and related Fc fusion proteins are currently reaching patent expiry allowing for the rise in biosimilar development [1]. Human Fc-fusion proteins have become a popular platform for biotherapeutics. Fusion proteins are typically constructed by fusing the Fc portion of human IgG1 with the extracellular domains of native transmembrane proteins linked to a target molecule [2]. Fusion proteins retain the function of the soluble receptor or ligand and have increased clinical performance due to the addition of the Fc portion of human IgG1, which allows for glycan-mediated clearance, improved protein stability and solubility, and IgG recycling for an extended half-life [3]. 

Glycosylation is an important post-translational protein modification process that alters protein properties including pharmacokinetics, effector functions, solubility, and stability [4,5,6]. Glycosylation is a critical quality attribute (CQA) for biosimilars, as slight differences in glycosylation patterns can induce immunogenicity. Consistent glycosylation is necessary to prove the drug’s safety and efficacy and to determine whether a biosimilar candidate is highly similar to the reference product. IgG monoclonal antibodies are known to have one conserved N-linked glycosylation site at Asn297 of the CH2 region of the Fc domain [4]. Fc-fusion proteins often carry several additional N-glycosylation sites in the non-IgG fusion protein part. The population of N-glycans occupying a particular site diverges by a complex series of modifications, which gives rise to structural heterogeneity or microheterogeneity. Such site-specific attributes require proper proteomic analysis for the characterization of glycoproteins.

Recent advances in mass spectrometry have allowed for high-throughput methods and accurate digitalized informatics for mapping glycosylation patterns [7,8,9]. A widely used analytical method for glycans released in IgG monoclonal antibodies is 2-AB labeling, in which the glycans are labeled with 2-aminobenzamide (2-AB) and analyzed by liquid chromatography equipped with fluorescent detection or by mass spectrometer [9,10]. 2-AB labeling can be advantageous due to its speed, reproducibility, cost-efficiency, and high sensitivity by fluorescence detection. While detection with 2-AB labeling may be a practical method for single-site glycan analysis in proteins such as IgG monoclonal antibodies, it is met with limitations for proteins that possess multiple glycosylation sites. During the labeling process, the release and pooling of detached glycans results in the loss of site-specific information on structural microheterogeneity for proteins with multiple sites of glycosylation, such as Fc fusion proteins [11].

To overcome these limitations, more sensitive characterization methods, such as liquid chromatography–electrospray ionization tandem mass spectrometry (LC-ESI MS/MS) can be used for site-specific characterization of complex glycoproteins that contain multiple N-glycosylation sites [12,13,14]. In this study, we prepared a VEGFR-IgG fusion protein comprising of a homodimer of vascular endothelial growth factor receptor 1 and 2 (VEGFR-1, VEGFR-2) fused to the Fc portion of human IgG1. The Fc fusion protein was expected to possess five N-linked glycosylation sites: two sites in the VEGFR-1 region, two sites in the VEGFR-2 region, and one site in the human IgG Fc region. LC-ESI MS/MS was applied for identifying N-linked glycopeptides and mapping site-specific glycosylation microheterogeneity of the prepared VEGFR-IgG fusion protein.

## 2. Results and Discussion

### 2.1. Cleaved Glycan Analysis by MALDI MS

**Figure 1:**
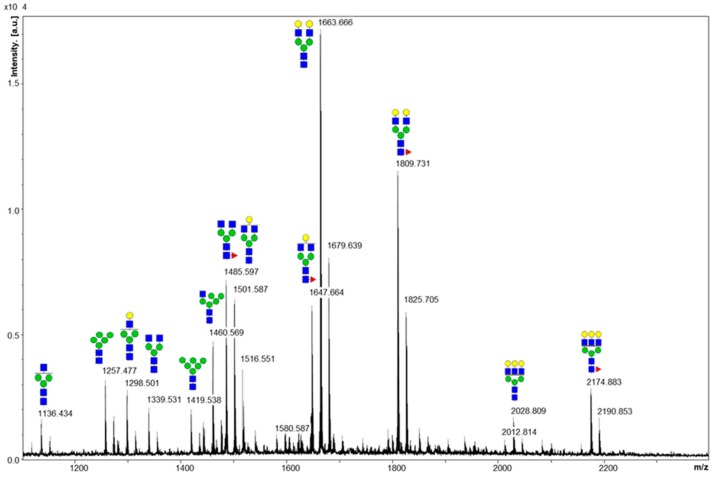
MALDI MS spectrum of the glycans released from the fusion protein. Sodium adducts of each glycan are depicted on the spectrum. The potassium adducts of glycans 5_4_0_0, 5_4_1_0, and 6_5_1_0 were also detected separately.

The biosimilar VEGFR-IgG fusion protein was developed by fusing the homodimer of VEGFR-1 and VEGFR-2 with the human IgG Fc region. The protein produced from fed-batch was desalted with a HiTrap Mabselect SuRe protein A column (GE Healthcare) according to manufacturer’s protocols and quantified by UV spectrometry and ELISA assay. The quantiifed protein was confirmed at the expected size of 57 kDA by SDS-PAGE and tested for possible impurities and aggregation by SEC-HPLC. Based on the resulting retention times, AU values, and peak area values obtained from SEC-HPLC, it was confirmed that the purified protein displayed minimal impurities and aggregation (data not shown). Glycan moieties of the fusion protein prepared by the affinity column were cleaved by using PNGase F and desalted by a carbon cartridge. The purified glycan sample was analyzed by a matrix-assisted laser desorption/ionization time-of-flight (MALDI-TOF) mass spectrometer. As shown in the MALDI-TOF mass spectrum (Figure 1), sodium adducts of glycans 3_4_0_0, 4_4_0_0, 4_4_1_0, 5_4_0_0 and 5_4_1_0 were detected as major peaks. In addition, we observed separate peaks for the potassium adducts of *glycans:* 5_4_0_0, 5_4_1_0, 6_5_1_0. Glycan nomenclature reflects the numbers of hexose (Hex), N-acetylglucosamine (GlcNAc), fucose (Fuc), and N-acetyl neuraminic acid (NeuAc) moieties (#Hex_#GlcNAc_#Fuc_#NeuAc). High mannose-type glycans 5_2_0_0, and 6_2_0_0 were also observed. Sialylated glycoforms of each glycans were not detected in the MALDI MS analysis, which may be due to the lability of sialic glycosyl linkage and/or low ionization efficiency of acidic glycans.

Unlike small molecule drugs, Fc fusion proteins are complex, heterogeneous proteins with multiple N-linked glycosylation sites resulting in vast site-specific heterogeneity, or glycan microheterogeneity. Although the presence of glycosylation on the fusion protein and the identification of major glycoforms can be accomplished by MALDI MS, our results displayed a noticeable limitation in the loss of site-specific information as this method can only provide information for the composition of total glycans pooled from each glycosylation site.

### 2.2. Protein Sequencing by LC-MS/MS

**Figure 2:**
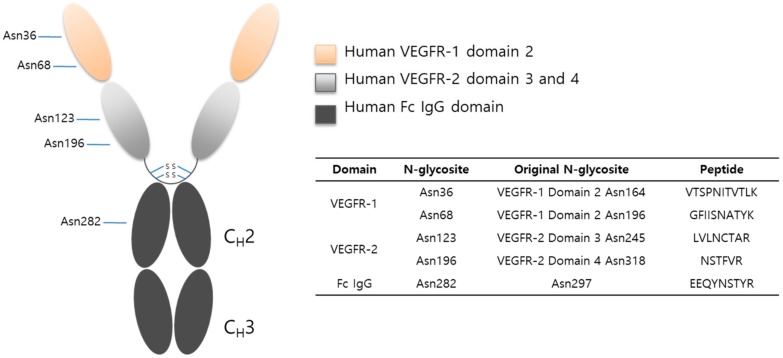
Schematic structure of VEGFR-IgG fusion protein.

The tryptic digests of VEGFR-IgG glycoprotein was desalted with an SPE micro-spin column and analyzed by LC-ESI MS/MS coupled with collision induced dissociation (CID) and high energy collision dissociation (HCD) fragmentation mode. The VEGFR-IgG fusion protein consisted of three regions: human VEGFR-1 domain 2, human VEGFR-2 domain 3 and 4, and human Fc IgG domain resulting in a total of five N-linked glycosylation sites (Figure 2).

**Figure 3:**
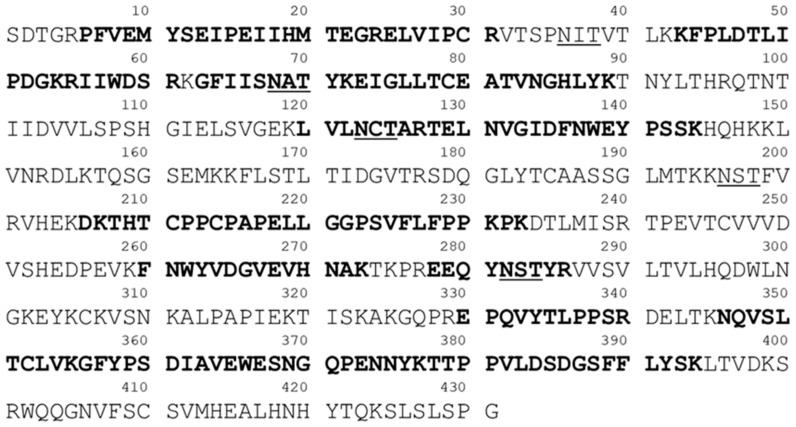
VEGFR-IgG fusion protein sequence. Bold characters present the identified sequences in the protein profiling.

As shown in Figure 3, 48.5% of the fusion protein sequence was identified by LC-ESI MS/MS analysis of the tryptic digests of VEGFR-IgG glycoprotein (The bolded peptides indicate the identified sequences). The peptide sequence identification was conducted under the following conditions: unlimited missed cleavage and 25 ppm tolerance of precursor ions. MS/MS spectra were assigned using the focused database of VEGFR-IgG protein, appending its reversed decoy sequence to increase the sequence coverage and the accuracy of the sequenced peptides. Final results of the identifed peptides showed false discovery rates (FDR) less than 0.01 (data not shown). It was also observed that some glycosylation sites were not fully occupied with N-glycans.

### 2.3. LC-MS/MS Glycopeptide Mapping of Fusion Protein

**Figure 4:**
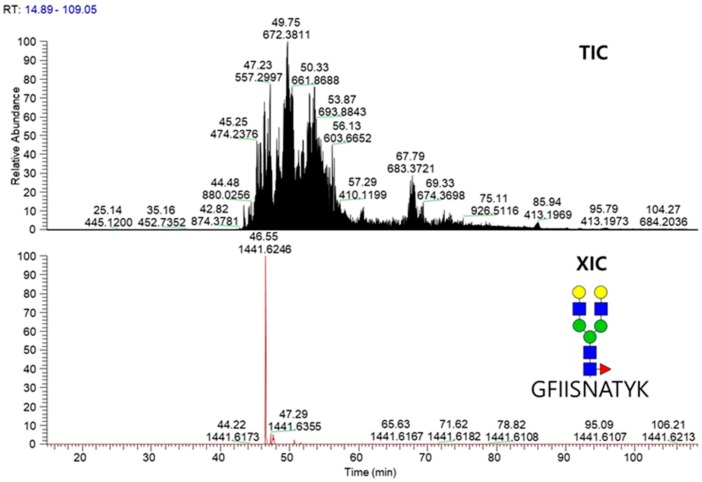
TIC and XIC chromatograms for glycopeptide GFIIS*N*ATYK_5_4_1_0 released from the fusion protein.

Glycopeptide mapping of VEGFR-IgG fusion protein was conducted using LC-ESI MS/MS coupled with CID and HCD fragmentation techniques. The VEGFR-1 region comprises VTSPNIITVTLK (Asn36) and GFIISNATYK (Asn68). LVLNCTAR (Asn123) and NSTFVR (Asn196) belongs to the VEGFR-2 region. The final site EEQYNSTYR (Asn282) is from the IgG1 Fc region, in which the site number corresponds to Asn297 on an intact IgG protein. Tryptic peptides with high complexity were first separated according to their hydrophobicity by liquid chromatography and N-glycopeptides well-separated by LC were then detected by online mass spectrometry. From the obtained tandem raw mass data, site-specific N-glycopeptides of VEGFR-IgG were automatically identified by Integrated GlycoProteome Analyzer (I-GPA) [15]. In the N-glycopeptide search using I-GPA, one target protein database was used for N-glycopeptide identification. Therefore, Y-score criteria ( > 60) instead of FDR was applied to filter out N-glycopeptides and N-glycopeptides filtered in were manually checked with criterias of retention times and isotopic mass distribution patterns. A total of 153 N-glycopeptides was identified from the five N-glycosylation sites of the fusion protein when one missed cleavage site was allowed for glycopeptide identification (Table S1). Figure 4 shows the total ion chromatogram (TIC) and extracted ion chromatogram (XIC) of glycopeptide GFIISNATYK_5_4_1_0 obtained from the analysis of VEGFR-IgG fusion proteins.

**Figure 5:**
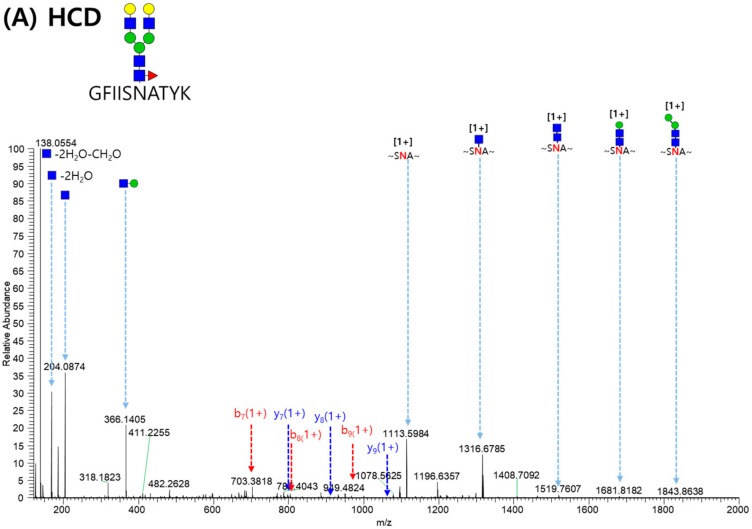

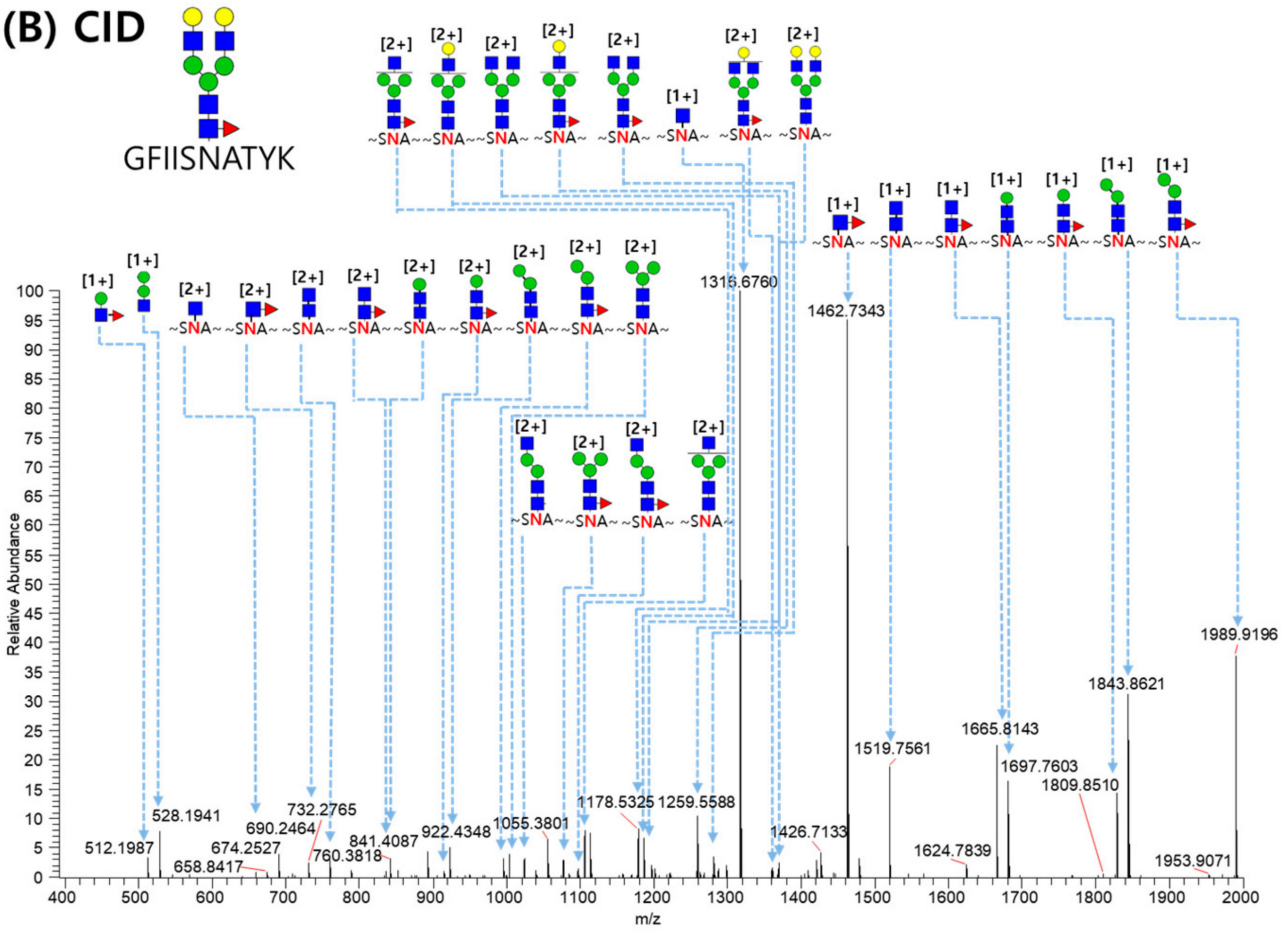
MS/MS spectrum of the GFIISNATYK_5_4_1_0 glycopeptide from the VEGFR-1 region (Asn68) matched automatically by GPA software. Spectrum (**A**) was obtained from HCD and spectrum (**B**) was obtained from CID.

Figure 5 displays the MS/MS spectrum of GFIISNATYK_5_4_1_0 glycopeptide from VEGFR-IgG (Asn68). Spectrums A and B were obtained from HCD and CID, respectively. The combination of HCD and CID fragmentation allows for the best identification of glycopeptides [16] because HCD and CID spectra provide information of the peptide sequence from b/y ions of peptide bond cleavage and of the glycan composition from glycan-cleaved glycopeptide fragment of glycoside bond cleavage, respectively. In HCD, high-energy fragmentation methods showed that oxonium ions and b/y ions were major, but B/Y ions were minor. On the contrary, *CID* low-energy fragmentation methods showed that B/Y ions were dominant, but oxonium ions and b/y ions were minor. In identifying the GFIISNATYK_5_4_1_0 glycopeptide, y_2_^+^ to y_9_^+^ and b_3_^+^ to b_9_^+^ ions (excluding b_6_^+^) were detected for peptide sequence information, and many glycan-cleaved glycopeptide fragment ions were detected for sufficient glycan composition determination.

**Figure 6:**
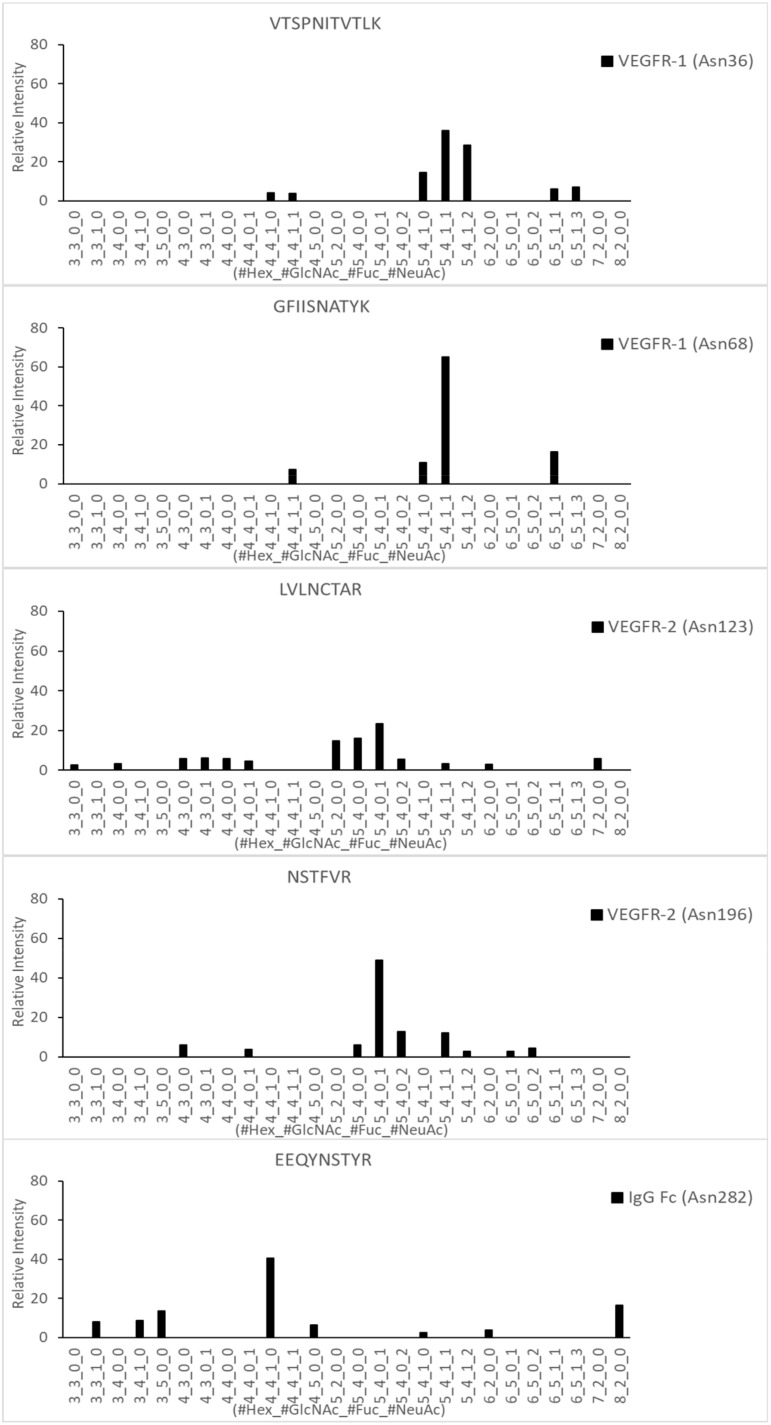
Site-specific N-glycopeptide mapping of the fusion protein. The relative abundance of the identified glycopeptides were normalized to each N-glycosylation site and less than 3% was cutoff.

The glycopeptide mapping results by LC-ESI MS/MS showed *noticeable* differences in the quantitative distribution of the most abundant N-linked glycoforms for the five N-glycosites (Figure 6). Site-specific features in glycosylation patterns could be observed as shown in Table 1. The relative abundance of fucosylation and sialylation in each N-linked glycopeptide identified from VEGFR-1, VEGFR-2, and IgG Fc regions were calculated based on the total sum of peak intensities for each N-glycosite. N-glycopeptides in the VEGFR-1 region (Asn36 and Asn68) showed 100% fucosylation ratios and over 80% were sialylation ratios, whereas the VEGFR-2 region displayed dramatically lower ratios of fucosylation (3.4% at Asn123 and 15.2% at Asn196) and significant sialylation ratios (42.7% at Asn123 and 87.8% at Asn196). However, N-glycopeptides from the IgG Fc region showed a 59.8% fucosylation ratio, and sialylated N-glycopeptides were not detected in this region.

As shown in the glycopeptide mapping data, two N-linked glycosylation sites (Asn36 and Asn68) in the VEGFR-1 portion of the fusion protein were observed to be highly galactosylated, fucosylated, and sialylated (VTSPNIITVTLK_5_4_1_1, VSTPNIITVTLK_5_4_1_2, VSTPNIITVTLK_6_5_1_1, VSTPNIITVTLK_6_5_1_3 on Asn36 and GFIISNATYK_4_4_1_1, GFIISNATYK_5_4_1_0, GFIISNATYK_5_4_1_1, GFIISNATYK_6_5_1_1 on Asn68). VEGFR-1 is known to be a receptor for proangiogenic ligands, VEGFA, VEGFB, and placental growth factor (PIGF), which have often been used in targeting therapies to slow tumor progression [17,18,19]. Previous studies have not only utilized the various IgG domains of VEGFR-1, but also introduced new glycosylation sites via mutagenesis in an attempt to alter the pharmacokinetic profile and increase binding affinity to three ligands: VEGFA, VEGFB, and PIGF. Such findings have shown that increased sialylation of VEGFR-1 can lead to higher binding affinity and is critical for in vivo half-life of proteins [20,21].

Two N-linked sites were also found in the VEGFR-2 portion of the fusion protein: Asn123 and Asn196 (Figure 6). Asn123 is found in domain 3 of VEGFR-2, which is reportedly known for VEGFR-2 ligand binding, and Asn196 is found in domain 4 of VEGFR-2, which has been reportedly involved in homodimer stabilization after ligand binding, receptor dimerization, and allosteric regulation of the receptor [22]. Asn123 displayed mostly sialylated glycoforms (LVLNCTAR_5_4_0_1, LVLNCTAR_5_4_0_2), as well as a high-mannose form (LVLNCTAR_5_2_0_0). Sialylated glycoforms were also detected in Asn196 (NSTFVR_5_4_0_1, NSTFVR_5_4_0_2, NSTFVR_5_4_1_1, NSTFVR_6_5_0_2). However, in comparison to the glycoforms detected in VEGFR-1, glycoforms found in VEGFR-2 displayed a low fucosylation ratio and a significant sialylation ratio. Interestingly, these glycosylation patterns for the VEGFR-2 domain displayed great differences with those obtained from a previous report, in which glycoprotein from the VEGFR-2 domain was produced by a murine cell line and presented mostly high mannose N-linked glycans with little indication of fully processed glycans [23]. These differences in glycosylation patterns may be due to the difference in the post-translational modification processes during protein sample preparation.

The Fc domain of the fusion protein displayed the major fucosylated glycoforms frequently found in human IgG (EEQYNSTYR_3_3_1_0, EEQYNSTYR_3_4_1_0, EEQYNSTYR_4_4_1_0, EEQYNSTYR_5_4_1_0 on Asn282) without sialylated glycoforms (Figure 6). Many studies have reported that over 90% N-glycopeptides of IgG from healthy human serum include core fucosylated structure [5,24,25,26,27]. The presence of high fucosylation is thought to provide regulation against potentially harmful antibody-dependent cell-mediated cytotoxicity (ADCC) activity in homeostasis [28]. It was also recently reported that the distribution of N-glycans in Fc fusion proteins may not be homogenous and requires domain-specific characterization for protein glycosylation mapping [29].

## 3. Materials and Methods

### 3.1. Materials

HiTrap Mabselect SuRe column was purchased from GE Healthcare (Chicago, IL, USA) and C18 trap column was purchased from Harvard Apparatus (Holliston, MA, USA). Trypsin for protein digestion was purchased from Promega (Madison, WI, USA). Phosphate buffered saline (PBS), 1,4-dithiothreitol (DTT), iodoacetamide (IAA), trifluoroacetic acid (TFA), ammonium bicarbonate (ABC), and formic acid (FA) were purchased from Sigma Aldrich (St. Louis, MO, USA). HPLC-grade water and acetonitrile (ACN) were purchased from J.T. Baker (Phillipsburg, NJ, USA). CHO-k1 cells were purchased from Sigma Aldrich.

### 3.2. Stable Cell Line Generation of the VEGFR-IgG Fusion Protein

Cell lines for the VEGFR-IgG fusion protein were generated using suspension-adapted CHO-k1 cells in chemically defined medium. In addition, 2 × 10^6^ cells were transfected by electroporation with 20 μg of the expression vector using a proprietary recombination-based transfection protocol. Transfected cells were monitored daily and subject to media change and antibiotic selection with puromycin dihydrochloride (10 μg/mL) every 48 hrs for 10–15 days. Stably transfected cells were selected for high-expressing clones with MoFlo-XDP (Beckman Coulter, Brea, CA, USA). Growth and specific productivity rates (SPR) were monitored by Clone Select Imager (CSI, Molecular Devices, San Jose, CA, USA) and by ELISA assay according to the protocol in Brezinksy et al. [30]. The final top three high-expressing clones were cryopreserved and tested under fed-batch conditions.

### 3.3. Stable Expression and Purification of VEGFR-IgG Fusion Protein

Fed-batch was performed by seeding 1.5 × 10^7^ cells in 30 mL growth medium and 10 μg/mL puromycin. Cells were supplemented with feeding medium every 48 h, maintained at 37 °C for 10–12 days and harvested when cell viability dropped below 75%. VEGFR-IgG fusion protein was purified from the cell culture supernatant using HiTrap Mabselect SuRe protein A columns and the resulting protein was subject to PBS buffer exchange, desalting and concentration using Amicon^®^ Ultra centrifugal filters (Merck Millipore, Burlington, MA, USA).

### 3.4. Cleaved Glycans Preparation

One hundred μL of protein sample (1 mg/mL) in PBS buffer, obtained from protein A affinity chromatography, was denatured by spiking 2-mercaptoethanol (1 M) and agitating for 2 min in a hot water bath (95 °C). The sample was diluted with ABC (200 mM) and spiked with 2 μL PNGase F (New England Biolab, UK). After incubation for 18 h at 37 °C, the glycan sample released from the protein was purified using an SPE PGC cartridge (Thermo Scientific, Waltham, MA, USA). The PGC cartridge was washed in advance with 80% ACN/0.1% TFA and equilibrated with water. The prepared sample was loaded on the PGC cartridge, washed with water, and eluted with 40% ACN/0.1% TFA. The eluted glycan sample was dried in a SpeedVac.

### 3.5. MALDI MS

Two μL of the glycan sample was dropped on the target plate and mixed with 2,5-dihydroxybenzoic acid (DHB) as a matrix. DHB was dissolved in acetonitrile/water (50:50, *v/v*) containing 0.5% TFA at a concentration of 10 mg/mL. The MS spectra were obtained using a Bruker UltrafleXtreme™ matrix-assisted laser desorption/ionization (MALDI) MS instrument (Bremen, Germany) equipped with a SmartBeam™ II laser.

### 3.6. Tryptic Digestion of Fusion Protein

One hundred μg of protein sample prepared by affinity column was denatured with ABC (50 mM) and urea (2 M). The sample was reduced by DTT (100 mM) for 1 hr at 35 °C. After incubation, the sample was alkylated with IAA (100 mM) for 1 hr in a darkroom and digested with trypsin (0.125 μg/ μL) overnight at 37 °C. The digested sample was dried in a SpeedVac and stored at −20 °C or reconstituted in 1% FA for desalting. SPE micro-spin column was equilibrated by centrifugation with wash buffer (99% H_2_O, 1% FA) and elution buffer (80% ACN, 1% FA). Aliquot of the tryptic digests was applied to the column, washed twice, and eluted twice with 30 μL elution buffer. The eluted peptide sample was dried in a SpeedVac.

### 3.7. LC-MS/MS Analysis

The digested sample was analyzed using an LC-MS/MS system consisting of Easy nLC 1200 (Thermo Fisher Scientific) and Orbitrap Fusion Lumos mass spectrometer (Thermo Fisher Scientific) equipped with a nano-electrospray source (EASY-Spray Sources, Thermo Fisher Scientific). Peptides were trapped in a 75 μm × 2 cm C_18_ precolumn (nanoViper, Acclaim PepMap100, Thermo Fisher Scientific) before being separated on an analytical C_18_ column (75 μm × 15 cm PepMap RSLC, Thermo Fisher Scientific) at a flow rate of 300 nL/min. The mobile phases A and B were composed of 0 and 80% acetonitrile containing 0.1% formic acid. The LC gradient began with 6% B for 1 min and was ramped to 25% B over 75 min, to 40% B over 15 min, to 100% B over 1 min, and remained at 100% B over 8 min, and 2% B for another 5 min. The column was re-equilibrated with 2% B for 15 min before the next run. The voltage applied to produce an electrospray was 1900 V. During the chromatographic separation, the Orbitrap Fusion Lumos was operated in data-dependent mode, automatically switching between MS1 and MS2 with 3 s of cycle time. The MS data were acquired using the following parameters: full scan MS1 spectral (400–2500 m/z) were acquired by Orbitrap for a maximum ion injection time of 100 ms at a resolution of 120,000 and an automatic gain control (AGC) target value of 4.0 × 10^5^. MS2 spectra were acquired by high energy collision dissociation (HCD) and collision induced dissociation (CID). The triggered condition was 2 detection of 3 (138.0545, 204.0867 and 366.1400 m/z) within the top 20 product ions. HCD and CID were performed in the Orbitrap mass analyzer at resolution of 30,000 with 35% normalized collision energy and AGC target value of 5.0 × 10^4^ with maximum ion injection time of 54 ms. Previously fragmented ions were excluded for 60 sec. 445.12003 m/z was used for the internal calibration.

### 3.8. N-Glycopeptide Identification

Site-specific VEGFR-IgG N-glycopeptides were automatically identified by an Integrated GlycoProteome Analyzer (I-GPA) [15]. In brief, N-glycopeptide tandem MS/MS spectra were first selected by using 15 glycan-specific oxonium reference ions. N-glycopeptide candidates were selected by matching their experimental MS isotope patterns to the theoretical patterns of N-glycopeptides in the GAP-DB, by combining possible tryptic peptides including N-glycosites of VEGFR-IgG, 351 N-glycans (331 retrosynthetic glycans from Kronewitter et al. [31]) and 20 glycans of penta and hexa polylactosamine series from Ozohanics et al. [32]. N-glycopeptides of VEGFR-IgG were identified by their Y-score upon matching of experimental and theoretical fragment ions. Y-scores were calculated by considering B/Y ions (oxonium ions and glycan cleaved glycopeptide fragment ions) from glycosidic bond cleavage of N-glycans attached on N-glycosylation sites and b/y ions (peptide backbone fragment ions) from peptide bond cleavage from HCD and CID ms/ms spectra. The common I-GPA search parameters were one missed cleavage, fixed modification of carbamidomethyl cysteine and mammalian N-glycan. The mass tolerances for precursor and for HCD fragment ions (CID) were set at 0.02 and 0.02 (1.5) Da, respectively. The Y-score was set above 60 instead of application of FDR < 0.05 in order to identify glycopeptides from a single targeted glycoprotein. Finally, we extracted uniquely identified N-glycopeptides with the highest Y-score and further checked manually with careful investigation in retention time and isotopic mass distribution pattern.

## 4. Conclusions

Fusion proteins are typically constructed by fusing the Fc portion of human IgG1 with extracellular domains of native transmembrane receptor proteins and often retain several N-glycosylation sites, including a single site at Asn297 in the Fc region of IgG1. The presence of multiple glycosylation sites in a glycoprotein requires a site-specific analysis method for full characterization of protein glycosylation. While conventional glycan-based methods, such as 2-AB labeling/fluorescence detection or mass analysis of glycans released from glycoprotein, are useful in identifying the glycoforms for monoclonal antibodies with single-site glycosylation, there are critical drawbacks in their ability to analyze glycoproteins with multiple glycosylation sites. In this study, the prepared VEGFR-Fc fusion consisting of VEGFR-1, VEGFR-2 and IgG Fc domain regions was trypsin-digested and analyzed through glycopeptide mapping using LC-ESI MS/MS. The expected five N-linked glycosylation sites were successfully identified and site-specific glycopeptide mapping was accomplished. Finally, it was clearly confirmed that N-linked glycans for each glycosylation site in the VEGFR-Fc fusion showed significantly different patterns in microheterogeneity. The distinctive glycosylation patterns in each glycosylation site may indicate the relevance of certain unknown functions of each domain in the fusion protein. Furthermore, the site-specific glycopeptide mapping strategy for fusion proteins with multiple glycosylation sites can be useful in future study designs to better understand the biological function of each receptor domain of the fusion protein.

## Figures and Tables

**Table 1:** Site-specific fucosylation and sialylation glycoform compositions in VEGFR-IgG fusion protein.

Domain	Site	Original Site	Peptide	Fuc%	Sia%
VEGFR-1	Asn57	VEGFR-1 Domain 2 Asn164	VTSPNITVTLK	100.0	81.6
	Asn90	VEGFR-1 Domain 2 Asn196	GFIISNATYK	100.0	89.0
VEGFR-2	Asn149	VEGFR-2 Domain 3 Asn245	LVLNCTAR	3.4	42.7
	Asn224	VEGFR-2 Domain 4 Asn318	NSTFVR	15.2	87.8
IgG Fc	Asn316	IgG Fc Domain Asn297	EEQYNSTYR	59.8	0.0

## Supplementary Materials

The following are available online at https://www.mdpi.com/1420-3049/24/21/3924/s1, Table S1: The list of site-specific N-glycpepitdes for VEGFR-IgG fusion protein identified by LC-ESI MS/MS analysis.
