# Supplementary material for: Investigation of Site-Specific Differences in Glycan Microheterogeneity by N-Glycopeptide Mapping of VEGFR-IgG Fusion Protein
Source: Molecules. 2019 Oct 30;24(21):3924. doi: 10.3390/molecules24213924 (PMC6864772; doi:10.3390/molecules24213924)
Supplement: Supplementary file 1 [file molecules-24-03924-s001.pdf]

Table S1: The list of site-specific N-glycpepitdes for VEGFR-IgG fusion protein identified by LC-ESI MS/MS analysis

RT	HCD_scan	M-score	MS_scan	Glycopeptides	Formula	CalMass	Delta_PPM	m/z	z	S-score	S-rank	HCD_score	CID_scan	CID_score	Y-score
44.16	6968	1.56	6967	EEQYNSTYR_3_3_1_0	H152C98O54N17	2430.9665	0.8536	1215.9882	2	99.99	1	81.96	6971	85.53	84.46
;	6978	1.6	6967	EEQYNSTYR_3_4_0_0	H155C100O55N18	2487.9879	2.3111	830.0031	3	99.96	1	62.35	6983	95.69	85.69
44.22	6990	1.5	6989	EEQYNSTYR_3_4_1_0	H165C106O59N18	2634.0458	0.9776	1317.5281	2	99.96	1	83.68	6993	84.39	84.18
44.13	6955	1.57	6945	EEQYNSTYR_3_5_0_0	H168C108O60N19	2691.0673	1.5421	897.6957	3	99.99	1	20.47	6959	78.71	61.24
44.12	6947	1.39	6945	EEQYNSTYR_4_4_1_0	H175C112O64N18	2796.0986	0.2772	1398.5536	2	99.93	1	67.9	6951	70.24	69.54
44.11	6946	1.46	6945	EEQYNSTYR_4_4_1_0	H175C112O64N18	2796.0986	5.9547	932.7103	3	99.95	1	73.96	6949	99.1	91.56
44.1	6938	1.46	6928	EEQYNSTYR_4_5_0_0	H178C114O65N19	2853.1201	1.2793	951.7107	3	99.97	1	25.8	6941	92.13	72.23
44.12	6948	1.3	6945	EEQYNSTYR_5_4_1_0	H185C118O69N18	2958.1515	0.9296	986.7233	3	99.97	1	75.74	6953	97.43	90.93
44.17	6970	1.29	6967	EEQYNSTYR_5_4_1_0	H185C118O69N18	2958.1515	1.1325	986.7235	3	99.04	1	77.56	6975	82.87	81.27
44.03	6912	1.51	6909	EEQYNSTYR_6_2_0_0	H159C102O60N16	2567.9876	0.9248	1284.4989	2	99.98	1	75.18	6917	75.09	75.12
44.02	6910	1.51	6909	EEQYNSTYR_7_2_0_0	H169C108O65N16	2730.0405	0.6135	1365.525	2	99.88	1	80.93	6913	72.01	74.69
44	6903	1.54	6892	EEQYNSTYR_8_2_0_0	H179C114O70N16	2892.0933	1.1324	1446.5522	2	99.93	1	74.14	6907	73.56	73.73
43.98	6895	1.61	6892	EEQYNSTYR_8_2_0_0	H179C114O70N16	2892.0933	1.4695	964.7044	3	99.95	1	77.27	6900	90.06	86.22
46.65	8024	1.34	8020	GFIISNATYK_4_4_1_0	H183C114O59N16	2720.1805	1.7922	1360.5966	2	99.88	1	69.02	8027	74.51	72.86
47.84	8546	1.64	8544	GFIISNATYK_4_4_1_1	H200C125O67N17	3011.2759	0.1162	1004.4304	3	99.82	1	76.33	8549	96.32	90.32
47.84	8547	1.67	8544	GFIISNATYK_4_4_1_1	H200C125O67N17	3011.2759	0.2242	1506.1422	2	99.94	1	70.76	8551	84.05	80.06
46.65	8026	1.55	8020	GFIISNATYK_5_2_0_0	H157C98O50N14	2330.0167	1.4056	1165.5139	2	99.95	1	74.07	8029	79.89	78.14
46.56	7986	1.27	7984	GFIISNATYK_5_4_1_0	H193C120O64N16	2882.2334	2.767	1441.6246	2	99.93	1	68.12	7989	84.93	79.89
47.71	8488	1.47	8486	GFIISNATYK_5_4_1_1	H210C131O72N17	3173.3288	1.0005	1587.1699	2	99.99	1	66.91	8492	86.44	80.58
47.7	8487	1.48	8486	GFIISNATYK_5_4_1_1	H210C131O72N17	3173.3288	1.5599	1058.4498	3	99.98	1	71.4	8490	98.46	90.34
47.62	8454	1.35	8451	GFIISNATYK_6_5_1_1	H233C145O82N18	3538.461	0.6712	1769.7356	2	99.79	1	74.54	8458	89.51	85.02
47.59	8444	1.41	8436	GFIISNATYK_6_5_1_1	H233C145O82N18	3538.461	1.2011	1180.1603	3	99.97	1	69.18	8449	100	90.75
54.32	11789	1.51	11785	GFIISNATYK_6_5_1_3	H267C167O98N20	4120.6518	2.1963	1374.2255	3	98.28	1	44.45	11792	91.68	77.51
45.5	7519	1.42	7516	KGFIISNATYK_3_3_1_0	H172C106O50N17	2483.1433	0.8658	828.3856	3	98.59	1	94.06	7523	53.22	65.47
45.43	7490	1.39	7480	KGFIISNATYK_4_4_1_0	H195C120O60N18	2848.2755	2.0539	950.099	3	99.94	1	66.04	7495	99.56	89.5
46.34	7889	1.54	7887	KGFIISNATYK_4_4_1_1	H212C131O68N19	3139.3709	3.6791	1047.125	3	99.45	1	65.78	7893	98.71	88.83
45.33	7446	1.29	7441	KGFIISNATYK_5_4_1_0	H205C126O65N18	3010.3283	2.4416	1004.1171	3	99.94	1	70.88	7451	98.86	90.47
46.26	7855	1.42	7852	KGFIISNATYK_5_4_1_1	H222C137O73N19	3301.4237	3.2486	1651.2104	2	99.53	1	64.23	7859	98.83	88.45
46.25	7853	1.43	7852	KGFIISNATYK_5_4_1_1	H222C137O73N19	3301.4237	3.3168	1101.1428	3	99.84	1	71.79	7856	98.66	90.6
47.36	8341	1.64	8334	KGFIISNATYK_5_4_1_2	H239C148O81N20	3592.5191	1.7119	1198.1803	3	99.96	1	69.87	8345	98.51	89.92
47.4	8360	1.53	8347	KGFIISNATYK_5_4_1_2	H239C148O81N20	3592.5191	2.6931	1796.7683	2	99.71	1	64.2	8364	99.09	88.62
46.31	7878	1.42	7869	KGFIISNATYK_5_5_0_1	H225C139O74N20	3358.4452	3.4986	1120.1497	3	98.87	1	15.22	7881	95.43	71.37
47.45	8375	1.64	8372	KGFIISNATYK_5_5_0_2	H242C150O82N21	3649.5406	0.2603	1217.1851	3	99.83	1	9.8	8379	95.53	69.81
45.3	7429	1.28	7422	KGFIISNATYK_6_5_1_0	H228C140O75N19	3375.4605	1.7923	1125.8274	3	99.82	1	63.65	7432	100	89.1
46.14	7801	1.39	7796	KGFIISNATYK_6_5_1_1	H245C151O83N20	3666.5559	0.2318	1222.8569	3	99.92	1	64.2	7806	100	89.26
46.1	7788	1.38	7782	KGFIISNATYK_7_6_1_1	H268C165O93N21	4031.6881	1.0541	1344.5665	3	99.34	1	70.45	7792	98.7	90.23
47.17	8261	1.41	8242	KGFIISNATYK_7_6_1_2	H285C176O101N22	4322.7835	3.6898	1441.5944	3	98.2	1	68.24	8264	89.27	82.96
48.98	9042	1.53	9035	KGFIISNATYK_7_6_1_3	H302C187O109N23	4613.879	2.5033	1538.6277	3	98.78	1	48.39	9045	95.1	81.09
41.18	5945	1.24	5944	KNSTFVR_3_3_0_0	H132C79O41N15	1946.8699	0.244	973.9391	2	99.85	1	100	5946	100	100
42.78	6438	1.26	6436	KNSTFVR_3_3_0_0	H132C79O41N15	1946.8699	0.4494	973.9393	2	99.89	1	94.03	6442	63.1	72.38
43.39	6688	1.34	6677	KNSTFVR_3_3_0_0	H132C79O41N15	1946.8699	0.4494	973.9393	2	99.89	1	75.91	6692	92.2	87.31
42.62	6371	1.47	6361	KNSTFVR_3_3_1_0	H142C85O45N15	2092.9278	0.0119	1046.9678	2	99.93	1	85.27	6374	97.66	93.94
42.62	6372	1.53	6361	KNSTFVR_3_3_1_0	H142C85O45N15	2092.9278	0.0239	698.3145	3	99.9	1	65.92	6376	72.07	70.23
42.74	6422	1.51	6416	KNSTFVR_3_3_1_0	H142C85O45N15	2092.9278	0.7406	698.315	3	99.78	1	84.16	6426	100	95.25
42.2	6196	1.5	6185	KNSTFVR_3_4_0_0	H145C87O46N16	2149.9493	0.0581	1075.4785	2	99.54	1	0	6200	100	70
42.36	6262	1.52	6259	KNSTFVR_3_4_0_0	H145C87O46N16	2149.9493	0.3488	717.3219	3	99.66	1	91.96	6267	98.48	96.52
41.81	6080	1.53	6079	KNSTFVR_3_4_0_0	H145C87O46N16	2149.9493	1.0465	717.3224	3	98.19	1	57.69	6081	91.52	81.37
42.49	6318	1.53	6314	KNSTFVR_3_4_1_0	H155C93O50N16	2296.0072	1.1542	766.0085	3	100	1	91.48			
42.58	6355	1.47	6348	KNSTFVR_3_4_1_0	H155C93O50N16	2296.0072	0.2504	1148.5078	2	99.89	1	58.92	6357	94.1	83.54
43.05	6547	1.48	6543	KNSTFVR_3_5_0_0	H158C95O51N17	2353.0287	1.1687	785.0157	3	99.94	1	54.41	6551	73.41	67.71
42.47	6306	1.46	6295	KNSTFVR_3_5_0_0	H158C95O51N17	2353.0287	1.5512	785.016	3	98	1	93.59	6310	93.93	93.83
42.52	6330	1.26	6329	KNSTFVR_4_3_0_0	H142C85O46N15	2108.9227	0.0593	1054.9652	2	99.97	1	95.96	6333	87.7	90.18
43.39	6687	1.33	6677	KNSTFVR_4_3_0_0	H142C85O46N15	2108.9227	0.0593	1054.9652	2	99.97	1	77.24	6690	89.43	85.78
42.49	6317	1.32	6314	KNSTFVR_4_3_0_0	H142C85O46N15	2108.9227	0.403	703.6464	3	99.97	1	98.13	6320	96.12	96.72
41.97	6122	1.27	6121	KNSTFVR_4_3_0_0	H142C85O46N15	2108.9227	0.4149	1054.9657	2	99.97	1	82.85	6123	88.51	86.81
43.32	6661	1.58	6658	KNSTFVR_4_3_0_1	H159C96O54N16	2400.0182	0.3229	1200.5134	2	100	1	78	6666	98.36	92.25
44.36	7046	1.46	7031	KNSTFVR_4_3_0_1	H159C96O54N16	2400.0182	1.5937	1200.5111	2	99.97	1	78.2	7048	86.79	84.21
43.31	6659	1.58	6658	KNSTFVR_4_3_0_1	H159C96O54N16	2400.0182	2.5625	800.68	3	99.99	1	83.97	6662	97.41	93.38
42.78	6439	1.31	6436	KNSTFVR_4_3_1_0	H152C91O50N15	2254.9806	0.0776	1127.9943	2	99.98	1	74.95	6444	89.98	85.47
43.64	6766	1.29	6760	KNSTFVR_4_3_1_0	H152C91O50N15	2254.9806	0.0776	1127.9943	2	99.98	1	100	6773	69.3	78.51
42.84	6464	1.33	6455	KNSTFVR_4_3_1_0	H152C91O50N15	2254.9806	0.1552	752.3322	3	99.9	1	88.54	6469	98.78	95.71
42.45	6298	1.32	6295	KNSTFVR_4_4_0_0	H155C93O51N16	2312.0021	0.0541	1156.5049	2	99.96	1	100	6303	99.02	99.32
42.6	6362	1.41	6361	KNSTFVR_4_4_0_0	H155C93O51N16	2312.0021	0.4542	771.3396	3	99.99	1	93.77	6365	100	98.13
42.04	6140	1.33	6139	KNSTFVR_4_4_0_0	H155C93O51N16	2312.0021	1.1029	771.3401	3	99.98	1	100	6141	100	100
41.53	6008	1.34	6007	KNSTFVR_4_4_0_0	H155C93O51N16	2312.0021	1.3625	771.3403	3	99.59	1	94.16	6009	100	98.25
43.49	6721	1.56	6718	KNSTFVR_4_4_0_1	H172C104O59N17	2603.0975	0.0288	1302.0527	2	99.87	1	91.9	6726	90.23	90.73
43.42	6699	1.67	6696	KNSTFVR_4_4_0_1	H172C104O59N17	2603.0975	1.3253	868.3722	3	99.99	1	82.13	6704	100	94.64
42.74	6421	1.32	6416	KNSTFVR_4_4_1_0	H165C99O55N16	2458.06	0.6407	1229.5347	2	99.89	1	78.49	6424	95.19	90.18
42.73	6417	1.43	6416	KNSTFVR_4_4_1_0	H165C99O55N16	2458.06	2.6647	820.0274	3	99.98	1	78.88	6419	100	93.66
43.64	6763	1.63	6760	KNSTFVR_4_4_1_1	H182C110O63N17	2749.1554	2.4917	917.0593	3	99.96	1	81.79	6767	99.64	94.29
42.78	6437	1.43	6436	KNSTFVR_4_5_0_0	H168C101O56N17	2515.0815	1.2127	839.0334	3	99.96	1	66.86	6440	92.27	84.65
42.18	6186	1.46	6185	KNSTFVR_4_5_0_0	H168C101O56N17	2515.0815	2.0476	839.0341	3	99.89	1	82.66	6189	94.58	91
42.25	6214	1.31	6204	KNSTFVR_4_5_1_0	H178C107O60N17	2661.1394	0.9582	887.7192	3	99.48	1	87.78	6217	85.62	86.27
42.91	6493	1.29	6491	KNSTFVR_4_5_2_0	H188C113O64N17	2807.1973	5.6818	936.399	3	99.79	1	90.47	6497	70.05	76.18
43.11	6577	1.34	6565	KNSTFVR_4_6_0_0	H181C109O61N18	2718.1609	0.6438	906.7261	3	98.05	1	40.54	6582	70.2	61.31
42.17	6180	1.47	6178	KNSTFVR_5_2_0_0	H139C83O46N14	2067.8962	0.5561	689.971	3	98.7	1	41.88	6183	96.36	80.01
43.36	6679	1.53	6677	KNSTFVR_5_3_0_1	H169C102O59N16	2562.071	1.6978	854.697	3	99.99	1	60.12	6683	65.39	63.81

42.68	6397	1.28	6396	KNSTFVR_5_4_0_0	H165C99O56N16	2474.0549	0.4547	1237.5308	2	100	1	93.7	6400	94.72	94.41
43.25	6633	1.28	6622	KNSTFVR_5_4_0_0	H165C99O56N16	2474.0549	0.4547	1237.5308	2	100	1	95.98	6637	82.85	86.79
42.82	6456	1.29	6455	KNSTFVR_5_4_0_0	H165C99O56N16	2474.0549	5.396	825.3613	3	99.96	1	86.23	6458	97.91	94.41
43.45	6708	1.44	6696	KNSTFVR_5_4_0_1	H182C110O64N17	2765.1504	0.1537	1383.0789	2	99.98	1	74.86	6714	98.81	91.62
44.4	7065	1.44	7052	KNSTFVR_5_4_0_1	H182C110O64N17	2765.1504	0.5244	922.3882	3	99.96	1	81.07	7068	85.36	84.07
44.33	7036	1.55	7031	KNSTFVR_5_4_0_2	H199C121O72N18	3056.2458	0.4172	764.817	4	99.99	1	91.06	7043	91.66	91.48
44.33	7033	1.55	7031	KNSTFVR_5_4_0_2	H199C121O72N18	3056.2458	0.5971	1528.6259	2	99.99	1	80.82	7037	97.88	92.76
43.75	6807	1.59	6800	KNSTFVR_5_4_0_2	H199C121O72N18	3056.2458	1.5869	1019.4221	3	99.98	1	81.25	6811	93.1	89.54
42.8	6446	1.27	6436	KNSTFVR_5_4_1_0	H175C105O60N16	2620.1128	0.1622	1310.5601	2	99.88	1	89.24	6449	90.77	90.31
42.01	6130	1.22	6128	KNSTFVR_5_4_1_0	H175C105O60N16	2620.1128	0.3626	874.0425	3	98.59	1	100	6133	94.06	95.84
42.56	6349	1.28	6348	KNSTFVR_5_4_1_0	H175C105O60N16	2620.1128	3.7594	874.0461	3	99.97	1	100	6351	95.53	96.87
43.14	6587	1.31	6584	KNSTFVR_5_4_1_0	H175C105O60N16	2620.1128	3.7594	874.0461	3	99.97	1	86.9	6592	94.31	92.08
43.64	6764	1.42	6760	KNSTFVR_5_4_1_1	H192C116O68N17	2911.2083	0.5754	1456.1089	2	99.97	1	89.16	6769	95.08	93.3
43.36	6678	1.56	6677	KNSTFVR_5_4_1_1	H192C116O68N17	2911.2083	5.9254	971.0804	3	99.9	1	69.25	6681	86.04	81.01
44.29	7014	1.6	7011	KNSTFVR_5_4_1_2	H209C127O76N18	3202.3037	0.2342	1068.1062	3	99.93	1	94.18	7018	94.27	94.24
42.08	6152	1.32	6151	KNSTFVR_5_4_2_0	H185C111O64N16	2766.1708	0.1988	922.729	3	99.54	1	78.13	6154	90.11	86.52
43.14	6586	1.66	6584	KNSTFVR_5_4_2_1	H202C122O72N17	3057.2662	0.7687	1019.7614	3	99.92	1	86.72	6590	97.74	94.43
42.96	6511	1.3	6510	KNSTFVR_5_5_0_0	H178C107O61N17	2677.1343	1.4755	893.0513	3	99.98	1	38.52	6514	97.38	79.72
43.48	6720	1.55	6718	KNSTFVR_5_5_0_1	H195C118O69N18	2968.2297	1.634	990.0834	3	99.98	1	52.46	6724	77.81	70.2
44.36	7047	1.69	7031	KNSTFVR_5_5_0_2	H212C129O77N19	3259.3251	1.6721	1087.1154	3	99.99	1	39.16	7050	94.17	77.67
42.44	6296	1.27	6295	KNSTFVR_5_5_1_0	H188C113O65N17	2823.1922	0.0531	941.736	3	98.67	1	61.83	6299	89.04	80.88
46.15	7808	1.54	7796	KNSTFVR_5_5_2_1	H215C130O77N18	3260.3455	2.5917	1087.4509	3	99.95	1	5.88	7812	94.03	67.59
43.23	6625	1.58	6622	KNSTFVR_6_3_0_1	H179C108O64N16	2724.1238	0.5047	1362.5665	2	99.93	1	92.6	6630	92.57	92.58
43.22	6623	1.67	6622	KNSTFVR_6_3_0_1	H179C108O64N16	2724.1238	2.2576	908.7152	3	99.99	1	84.48	6626	96.93	93.2
41.9	6104	1.28	6103	KNSTFVR_6_4_0_0	H175C105O61N16	2636.1078	0.019	879.3745	3	99.65	1	100	6105	98.11	98.68
43.28	6643	1.53	6641	KNSTFVR_6_4_0_1	H192C116O69N17	2927.2032	1.2469	976.4075	3	99.97	1	87.86	6645	98.73	95.46
42.11	6160	1.25	6159	KNSTFVR_6_5_0_0	H188C113O66N17	2839.1871	0.2289	947.0678	3	99.94	1	55.55	6162	83.88	75.38
42.68	6398	1.31	6396	KNSTFVR_6_5_0_0	H188C113O66N17	2839.1871	0.405	947.0672	3	99.97	1	94.62	6402	94.4	94.46
43.52	6729	1.46	6728	KNSTFVR_6_5_0_1	H205C124O74N18	3130.2825	0.7827	1044.1002	3	99.98	1	94.26	6732	94.89	94.7
43.23	6624	1.33	6622	KNSTFVR_6_5_0_1	H205C124O74N18	3130.2825	1.3657	1565.6473	2	99.94	1	79.07	6628	98.76	92.85
44.17	6969	1.52	6967	KNSTFVR_6_5_0_2	H222C135O82N19	3421.378	1.213	1141.1326	3	99.99	1	95.27	6973	97.52	96.85
42.36	6260	1.26	6259	KNSTFVR_6_5_1_0	H198C119O70N17	2985.245	0.1507	995.7537	3	99.98	1	100	6263	98.43	98.9
43.04	6546	1.34	6543	KNSTFVR_6_5_1_0	H198C119O70N17	2985.245	0.2512	995.7538	3	99.99	1	90.47	6549	99.24	96.61
43.07	6558	1.43	6543	KNSTFVR_6_5_1_1	H215C130O78N18	3276.3405	0.4731	1092.7859	3	99.97	1	83.95	6563	99.73	94.99
44.38	7055	1.58	7052	KNSTFVR_6_5_1_2	H232C141O86N19	3567.4359	0.6868	1189.818	3	99.99	1	80.14	7058	98.33	92.87
42.87	6475	1.25	6471	KNSTFVR_6_6_0_0	H201C121O71N18	3042.2665	0.0164	1014.7607	3	99.99	1	46.9	6480	80.94	70.73
43.39	6689	1.38	6677	KNSTFVR_6_6_0_1	H218C132O79N19	3333.3619	2.265	1111.79	3	99.62	1	28.57	6694	92.95	73.64
42.46	6305	1.3	6295	KNSTFVR_7_4_0_0	H185C111O66N16	2798.1606	0.1251	933.3922	3	99.89	1	31.67	6308	88.4	71.38
43.32	6660	1.5	6658	KNSTFVR_7_4_0_1	H202C122O74N17	3089.256	1.0844	1030.425	3	99.95	1	82.61	6664	95.97	91.96
42.64	6381	1.3	6380	KNSTFVR_7_6_0_0	H211C127O76N18	3204.3193	1.1079	1068.7795	3	99.68	1	83.99	6384	100	95.2
43.29	6649	1.44	6641	KNSTFVR_7_6_0_1	H228C138O84N19	3495.4147	0.329	1165.8105	3	99.98	1	82.55	6652	98.93	94.02
44.1	6940	1.49	6928	KNSTFVR_7_6_0_2	H245C149O92N20	3786.5102	1.4657	1262.8438	3	99.92	1	83.72	6943	97.87	93.62
44.98	7295	1.4	7290	LVLNCTAR_3_2_0_0	H128C73S1O37N15	1838.831	0.5302	919.9199	2	99.99	1	89.31	7300	78.45	81.71
44.96	7283	1.55	7271	LVLNCTAR_3_3_0_0	H141C81S1O42N16	2041.9104	0.2081	1021.4589	2	100	1	89.98	7288	88.37	88.86
44.98	7293	1.52	7290	LVLNCTAR_3_4_0_0	H154C89S1O47N17	2244.9898	0.2561	1122.9991	2	99.95	1	81.03	7296	89.92	87.26
44.98	7294	1.58	7290	LVLNCTAR_3_4_0_0	H154C89S1O47N17	2244.9898	0.6459	749.0023	3	99.88	1	78.13	7298	73.18	74.67
44.9	7254	1.33	7251	LVLNCTAR_4_3_0_0	H151C87S1O47N16	2203.9632	0.2836	1102.4852	2	99.99	1	76.1	7259	91.07	86.58
45.97	7742	1.31	7728	LVLNCTAR_4_3_0_0	H151C87S1O47N16	2203.9632	0.6239	1102.4862	2	99.97	1	72.81	7743	87.29	82.95
44.96	7282	1.32	7271	LVLNCTAR_4_3_0_0	H151C87S1O47N16	2203.9632	0.8394	735.3269	3	99.99	1	86.09	7286	95.2	92.47
46	7753	1.51	7745	LVLNCTAR_4_3_0_1	H168C98S1O55N17	2495.0586	2.3847	832.3561	3	99.87	1	91.15	7756	97.37	95.5
45.98	7746	1.64	7745	LVLNCTAR_4_3_0_1	H168C98S1O55N17	2495.0586	3.056	1248.0294	2	99.97	1	89.35	7749	93.99	92.6
44.94	7273	1.35	7271	LVLNCTAR_4_4_0_0	H164C95S1O52N17	2407.0426	0.592	1204.0245	2	99.99	1	93.64	7276	90.37	91.35
44.94	7274	1.39	7271	LVLNCTAR_4_4_0_0	H164C95S1O52N17	2407.0426	1.4748	803.0206	3	99.99	1	94.02	7278	97.66	96.57
45.94	7731	1.68	7728	LVLNCTAR_4_4_0_1	H181C106S1O60N18	2698.138	2.5295	1349.5695	2	99.97	1	72.44	7735	91.02	85.45
45	7304	1.46	7290	LVLNCTAR_4_4_1_0	H174C101S1O56N17	2553.1005	1.0771	851.7063	3	99.89	1	73.03	7309	82.02	79.33
46.01	7759	1.6	7745	LVLNCTAR_4_4_1_1	H191C112S1O64N18	2844.1959	1.4327	1422.6039	2	99.39	1	92.23	7760	89.71	90.47
44.87	7245	1.5	7235	LVLNCTAR_5_2_0_0	H148C85S1O47N15	2162.9367	3.5484	1081.9761	2	99.99	1	82.8	7249	83.1	83.01
45.95	7734	1.58	7728	LVLNCTAR_5_3_0_1	H178C104S1O60N17	2657.1115	1.3737	886.3745	3	99.9	1	76.81	7739	92.19	87.58
45.94	7732	1.67	7728	LVLNCTAR_5_3_0_1	H178C104S1O60N17	2657.1115	2.0793	1329.0569	2	99.98	1	90.19	7737	88.32	88.88
44.9	7253	1.29	7251	LVLNCTAR_5_4_0_0	H174C101S1O57N17	2569.0954	0.3017	1285.052	2	99.99	1	93.17	7257	89.66	90.71
44.89	7252	1.27	7251	LVLNCTAR_5_4_0_0	H174C101S1O57N17	2569.0954	5.5856	857.0418	3	99.95	1	84.42	7255	96.64	92.98
45.9	7710	1.42	7708	LVLNCTAR_5_4_0_1	H191C112S1O65N18	2860.1908	0.6118	954.0694	3	100	1	91.91	7714	97.7	95.96
45.89	7709	1.43	7708	LVLNCTAR_5_4_0_1	H191C112S1O65N18	2860.1908	1.2499	1430.6011	2	99.96	1	88.57	7712	91.67	90.74
47.34	8338	1.65	8334	LVLNCTAR_5_4_0_2	H208C123S1O73N19	3151.2863	1.9278	1576.1501	2	99.96	1	71.83	8342	92.28	86.14
47.39	8352	1.61	8347	LVLNCTAR_5_4_0_2	H208C123S1O73N19	3151.2863	2.0468	1051.1028	3	99.97	1	93.48	8356	93.78	93.69
44.92	7261	1.31	7251	LVLNCTAR_5_4_1_0	H184C107S1O61N17	2715.1533	0.2394	905.7232	3	99.83	1	90.5	7265	95.85	94.24
45.85	7691	1.43	7685	LVLNCTAR_5_4_1_1	H201C118S1O69N18	3006.2487	1.0811	1002.7537	3	99.87	1	78.07	7694	98.14	92.12
45.85	7693	1.46	7685	LVLNCTAR_5_4_1_1	H201C118S1O69N18	3006.2487	1.1726	1503.6265	2	99.87	1	68.95	7696	90.35	83.93
47.41	8362	1.49	8347	LVLNCTAR_5_4_1_2	H218C129S1O77N19	3297.3442	0.7127	1099.7874	3	99.36	1	71.74	8368	84.48	80.66
44.85	7236	1.49	7235	LVLNCTAR_6_2_0_0	H158C91S1O52N15	2324.9895	0.2903	1162.999	2	99.99	1	88.3	7239	83.84	85.18
44.87	7244	1.35	7235	LVLNCTAR_6_3_0_0	H171C99S1O57N16	2528.0689	1.1372	1264.5398	2	99.99	1	75.86	7247	73.46	74.18
44.86	7238	1.4	7235	LVLNCTAR_6_3_0_0	H171C99S1O57N16	2528.0689	3.3029	843.3643	3	99.89	1	84.06	7241	94.92	91.66
45.9	7711	1.66	7708	LVLNCTAR_6_3_0_1	H188C110S1O65N17	2819.1643	0.807	1410.0872	2	99.82	1	90.21	7716	80.39	83.34
45.92	7721	1.62	7708	LVLNCTAR_6_3_0_1	H188C110S1O65N17	2819.1643	1.4011	940.392	3	99.78	1	80.07	7726	95.16	90.63
44.81	7218	1.52	7217	LVLNCTAR_7_2_0_0	H168C97S1O57N15	2487.0423	1.156	1244.0265	2	100	1	89.46	7221	80.92	83.48
44.83	7226	1.65	7217	LVLNCTAR_7_2_0_0	H168C97S1O57N15	2487.0423	1.9501	829.6876	3	99.96	1	98.97	7231	81.19	86.53
43.6	6749	1.47	6738	NSTFVR_3_3_0_0	H120C73O40N13	1818.775	2.2955	909.8935	2	99.99	1	72.09	6754	66.85	68.42
44.51	7109	1.45	7096	NSTFVR_3_3_0_0	H120C73O40N13	1818.775	3.6151	909.8947	2	99.96	1	73.19	7113	90.13	85.05
42.69	6399	1.4	6396	NSTFVR_4_2_0_0	H117C71O40N12	1777.7484	2.5735	889.3804	2	99.93	1	100	6404	72.63	80.84
44.48	7099	1.36	7096	NSTFVR_4_3_0_0	H130C79O45N13	1980.8278	0.5932	990.9184	2	99.97	1	70.05	7104	89.31	83.53
44.54	7119	1.57	7117	NSTFVR_4_3_0_1	H147C90O53N14	2271.9232	0.3411	1136.4659	2	99.99	1	71.26	7123	92.15	85.88

43.7	6788	1.34	6778	NSTFVR_4_3_1_0	H140C85O49N13	2126.8857	1.2577	1063.9481	2	99.97	1	62.47	6792	66.96	65.61
44.56	7128	1.73	7117	NSTFVR_4_4_0_1	H160C98O58N15	2475.0026	0.0101	1238.0052	2	99.75	1	89.82	7132	86.53	87.52
44.54	7120	1.67	7117	NSTFVR_4_4_0_1	H160C98O58N15	2475.0026	1.8788	825.6743	3	99.98	1	76.59	7125	98.78	92.13
44.63	7154	1.59	7152	NSTFVR_4_4_1_1	H170C104O62N15	2621.0605	2.0412	874.3605	3	99.97	1	94.17	7157	86.85	89.05
42.58	6356	1.48	6348	NSTFVR_5_2_0_0	H127C77O45N12	1939.8012	0.915	970.4054	2	99.99	1	100	6359	89.13	92.39
43.13	6585	1.39	6584	NSTFVR_5_2_0_0	H127C77O45N12	1939.8012	1.3275	970.4058	2	100	1	80.91	6588	76.88	78.09
43.34	6668	1.38	6658	NSTFVR_5_3_0_0	H140C85O50N13	2142.8806	0.5483	1071.9448	2	99.45	1	84.55	6671	74.41	77.45
44.51	7108	1.56	7096	NSTFVR_5_3_0_1	H157C96O58N14	2433.976	1.1401	1217.4933	2	99.99	1	68.06	7111	73.61	71.94
43.57	6739	1.28	6738	NSTFVR_5_4_0_0	H153C93O55N14	2345.96	0.5861	1173.4846	2	100	1	92.03	6742	90.12	90.69
43.58	6740	1.31	6738	NSTFVR_5_4_0_0	H153C93O55N14	2345.96	3.5167	782.6613	3	99.98	1	79.77	6744	94.22	89.88
44.48	7098	1.53	7096	NSTFVR_5_4_0_1	H170C104O63N15	2637.0554	0.6731	1319.0325	2	100	1	64.82	7102	93.23	84.71
44.48	7097	1.5	7096	NSTFVR_5_4_0_1	H170C104O63N15	2637.0554	2.2184	879.6923	3	99.99	1	75.29	7100	97.96	91.16
45.87	7700	1.59	7685	NSTFVR_5_4_0_2	H187C115O71N16	2928.1508	0.7599	1464.5782	2	99.98	1	61.37	7704	91.5	82.46
45.87	7699	1.56	7685	NSTFVR_5_4_0_2	H187C115O71N16	2928.1508	2.4077	976.7198	3	99.92	1	69.63	7702	96.43	88.39
43.68	6780	1.29	6778	NSTFVR_5_4_1_0	H163C99O59N14	2492.0179	0.4314	1246.5134	2	99.98	1	92.79	6784	86.04	88.07
43.68	6779	1.33	6778	NSTFVR_5_4_1_0	H163C99O59N14	2492.0179	3.2303	831.3472	3	99.99	1	77.26	6782	95.14	89.78
44.58	7137	1.43	7136	NSTFVR_5_4_1_1	H180C110O67N15	2783.1133	0.6018	1392.0614	2	100	1	94.44	7140	90.39	91.6
44.45	7085	1.5	7074	NSTFVR_5_4_1_1	H180C110O67N15	2783.1133	6.0184	928.3819	3	99.94	1	92.75	7090	97.89	96.35
45.81	7677	1.59	7661	NSTFVR_5_4_1_2	H197C121O75N16	3074.2087	0.4961	1537.6075	2	99.5	1	62.81	7680	91.05	82.57
45.82	7682	1.55	7661	NSTFVR_5_4_1_2	H197C121O75N16	3074.2087	1.7403	1025.4099	3	99.83	1	53.28	7683	84.46	75.11
42.34	6254	1.56	6239	NSTFVR_6_2_0_0	H137C83O50N12	2101.854	0.0119	1051.4309	2	100	1	97.7	6257	87.4	90.49
42.91	6492	1.57	6491	NSTFVR_6_2_0_0	H137C83O50N12	2101.854	0.0119	1051.4309	2	100	1	90.95	6495	76.83	81.07
43.25	6632	1.39	6622	NSTFVR_6_3_0_0	H150C91O55N13	2304.9334	0.6833	1152.9714	2	99.97	1	89.87	6635	87.87	88.47
43.29	6651	1.41	6641	NSTFVR_6_3_0_0	H150C91O55N13	2304.9334	1.6703	768.9843	3	99.85	1	79.26	6656	72.9	74.81
43.28	6644	1.28	6641	NSTFVR_6_5_0_0	H176C107O65N15	2711.0922	2.0103	904.3711	3	99.98	1	65.11	6647	94.6	85.75
44.4	7066	1.44	7052	NSTFVR_6_5_0_1	H193C118O73N16	3002.1876	0.7411	1501.5966	2	99.81	1	86.96	7070	79.1	81.46
44.33	7035	1.39	7031	NSTFVR_6_5_0_1	H193C118O73N16	3002.1876	2.0152	1001.4031	3	99.94	1	73.83	7041	98.69	91.23
45.63	7585	1.53	7577	NSTFVR_6_5_0_2	H210C129O81N17	3293.283	0.3796	1098.4333	3	99.99	1	73	7588	97.91	90.44
45.68	7608	1.45	7600	NSTFVR_6_5_0_2	H210C129O81N17	3293.283	0.9641	1647.147	2	99.8	1	90.2	7612	95.17	93.68
44.38	7056	1.4	7052	NSTFVR_6_5_1_1	H203C124O77N16	3148.2455	1.0958	1050.0859	3	99.99	1	71.82	7060	98.88	90.76
45.76	7651	1.52	7643	NSTFVR_6_5_1_2	H220C135O85N17	3439.3409	0.0436	1147.1189	3	99.67	1	64.29	7654	90.66	82.75
45.76	7649	1.48	7643	NSTFVR_6_5_1_2	H220C135O85N17	3439.3409	1.6064	1720.1716	2	98.56	1	94.45	7652	89.73	91.15
42.28	6228	1.52	6221	NSTFVR_7_2_0_0	H147C89O55N12	2263.9069	0.1436	1132.4572	2	99.97	1	93.07	6231	81.26	84.8
43.05	6548	1.51	6543	NSTFVR_7_2_0_0	H147C89O55N12	2263.9069	0.1436	1132.4572	2	99.97	1	100	6553	83.83	88.68
42.76	6430	1.3	6416	NSTFVR_8_2_0_0	H157C95O60N12	2425.9597	1.4324	1213.4855	2	99.88	1	89.79	-	-	-
42.42	6288	1.48	6278	NSTFVR_9_2_0_0	H167C101O65N12	2588.0125	1.8257	1294.5078	2	99.35	1	92.73	6293	55.14	66.42
43.42	6698	1.56	6696	TKPREEQYNSTYR_3_4_0_0	H193C121O60N26	2970.2844	2.6008	743.3289	4	99.54	1	76.89	6702	100	93.07
43.42	6697	1.61	6696	TKPREEQYNSTYR_3_4_1_0	H203C127O64N26	3116.3423	0.714	779.842	4	99.95	1	83.41	6700	100	95.02
43.44	6706	1.61	6696	TKPREEQYNSTYR_3_5_0_0	H206C129O65N27	3173.3638	2.2453	794.0986	4	99.98	1	48.82	6710	98.8	83.8
43.74	6802	1.43	6800	TKPREEQYNSTYR_4_5_0_0	H216C135O70N27	3335.4166	1.6565	834.6114	4	99.93	1	44.11	6804	98.75	82.36
43.37	6680	1.52	6677	TKPREEQYNSTYR_5_2_0_0	H187C117O60N24	2888.2313	0.9781	722.8144	4	99.09	1	78.57	6685	64.07	68.42
43.45	6709	1.28	6696	TKPREEQYNSTYR_5_4_1_0	H223C139O74N26	3440.448	0.0363	860.8679	4	99.86	1	84.86	6716	97.09	93.42
43.34	6669	1.56	6658	TKPREEQYNSTYR_6_2_0_0	H197C123O65N24	3050.2842	1.4179	763.328	4	99.79	1	87.11	6673	88.94	88.39
46.94	8148	1.46	8143	VTSPNITVTLK_3_2_1_0	H160C92O46N15	2211.0636	0.8254	1106.0348	2	99.85	1	75.9	8152	79.43	78.37
46.9	8129	1.43	8126	VTSPNITVTLK_3_3_1_0	H173C100O51N16	2414.143	2.5786	1207.5723	2	99.48	1	61.78	8133	76.86	72.34
46.87	8117	1.56	8111	VTSPNITVTLK_3_4_1_0	H186C108O56N17	2617.2223	1.8053	1309.1127	2	99.81	1	71.59	8122	90.4	84.76
48.25	8717	1.59	8707	VTSPNITVTLK_4_3_1_1	H200C117O64N17	2867.2912	1.9443	1434.1523	2	99.71	1	73.65	8720	86.5	82.65
46.83	8098	1.35	8094	VTSPNITVTLK_4_4_1_0	H196C114O61N17	2779.2752	0.8006	1390.1404	2	99.95	1	74.36	8101	91.28	86.21
48.12	8668	1.64	8658	VTSPNITVTLK_4_4_1_1	H213C125O69N18	3070.3706	0.9852	1535.6877	2	99.94	1	70.95	8671	91.97	85.67
48.07	8651	1.63	8644	VTSPNITVTLK_4_4_1_1	H213C125O69N18	3070.3706	2.003	1024.1267	3	98.4	1	72.94	8654	90.93	85.53
46.87	8116	1.53	8111	VTSPNITVTLK_5_2_0_0	H170C98O52N15	2389.1113	0.2197	1195.0593	2	99.96	1	73.79	8120	81.94	79.49
46.79	8077	1.29	8075	VTSPNITVTLK_5_4_1_0	H206C120O66N17	2941.328	0.2635	1471.1683	2	99.97	1	74.13	8080	93.32	87.57
46.83	8099	1.21	8094	VTSPNITVTLK_5_4_1_0	H206C120O66N17	2941.328	0.357	981.1142	3	99.92	1	54.16	8103	91.1	80.02
48.1	8659	1.45	8658	VTSPNITVTLK_5_4_1_1	H223C131O74N18	3232.4234	1.3071	1616.7135	2	99.91	1	76.22	8662	95.71	89.87
48.67	8902	1.47	8891	VTSPNITVTLK_5_4_1_1	H223C131O74N18	3232.4234	1.3071	1616.7135	2	99.91	1	75.66	8905	90.81	86.27
47.92	8582	1.47	8579	VTSPNITVTLK_5_4_1_1	H223C131O74N18	3232.4234	1.9026	1078.1484	3	99.97	1	78.86	8586	100	93.66
50.98	10016	1.58	10003	VTSPNITVTLK_5_4_1_2	H240C142O82N19	3523.5188	0.149	881.6357	4	98.89	1	16.73	10021	86.17	65.34
51.66	10328	1.66	10319	VTSPNITVTLK_5_4_1_2	H240C142O82N19	3523.5188	0.8089	1175.1772	3	99.86	1	74.32	10331	94.34	88.33
50.11	9591	1.64	9581	VTSPNITVTLK_5_4_1_2	H240C142O82N19	3523.5188	0.9791	1175.177	3	99.73	1	91.52	9595	87.6	88.78
50.15	9610	1.66	9599	VTSPNITVTLK_5_4_1_2	H240C142O82N19	3523.5188	0.9791	1175.177	3	99.73	1	91.58	9613	89.55	90.16
50.49	9769	1.65	9768	VTSPNITVTLK_5_4_1_2	H240C142O82N19	3523.5188	0.9791	1175.177	3	99.73	1	88.34	9772	96.8	94.26
51.09	10059	1.64	10055	VTSPNITVTLK_5_4_1_2	H240C142O82N19	3523.5188	0.9791	1175.177	3	99.73	1	76.07	10063	99.23	92.28
51.39	10194	1.63	10189	VTSPNITVTLK_5_4_1_2	H240C142O82N19	3523.5188	1.2417	1762.2655	2	99.78	1	67.69	10198	94.1	86.18
51.9	10446	1.56	10427	VTSPNITVTLK_5_4_1_2	H240C142O82N19	3523.5188	1.2417	1762.2655	2	99.85	1	53.99	10449	83.29	74.5
50.82	9935	1.65	9934	VTSPNITVTLK_5_4_1_2	H240C142O82N19	3523.5188	1.7099	1762.2603	2	99.65	1	68.99	9938	96.61	88.32
64.77	16616	1.58	16600	VTSPNITVTLK_5_4_1_2	H240C142O82N19	3523.5188	3.6185	1175.1824	3	99.85	1	91.69	16619	94.05	93.34
51.35	10172	1.65	10168	VTSPNITVTLK_5_5_0_2	H243C144O83N20	3580.5403	0.4888	1194.1859	3	99.77	1	11.75	10176	88.6	65.55
50.62	9834	1.67	9825	VTSPNITVTLK_5_5_0_2	H243C144O83N20	3580.5403	1.0194	1194.1841	3	98.88	1	11.31	9837	89.17	65.81
50.74	9900	1.64	9892	VTSPNITVTLK_5_5_0_2	H243C144O83N20	3580.5403	1.0194	1194.1841	3	98.88	1	21.8	9902	90.06	69.58
46.74	8059	1.22	8056	VTSPNITVTLK_6_5_1_0	H229C134O76N18	3306.4602	0.1739	1653.7343	2	99.76	1	67.04	8063	85.01	79.62
47.9	8572	1.37	8562	VTSPNITVTLK_6_5_1_1	H246C145O84N19	3597.5556	0.6046	1799.2828	2	99.92	1	71.69	8575	91.99	85.9
47.88	8564	1.38	8562	VTSPNITVTLK_6_5_1_1	H246C145O84N19	3597.5556	1.2647	1199.8586	3	99.99	1	77.91	8567	99.63	93.11
50.02	9552	1.5	9543	VTSPNITVTLK_6_5_1_2	H263C156O92N20	3888.651	0.2957	1296.8885	3	99.11	1	68.37	9555	99.63	90.25
50.42	9737	1.51	9733	VTSPNITVTLK_6_5_1_2	H263C156O92N20	3888.651	0.2957	1296.8885	3	99.11	1	68.65	9740	99.45	90.21
50.66	9854	1.52	9848	VTSPNITVTLK_6_5_1_2	H263C156O92N20	3888.651	0.2957	1296.8885	3	99.11	1	71.71	9858	98.75	90.64
51.06	10045	1.52	10039	VTSPNITVTLK_6_5_1_2	H263C156O92N20	3888.651	0.2957	1296.8885	3	99.11	1	67.91	10049	93.95	86.14
50.39	9725	1.52	9718	VTSPNITVTLK_6_5_1_2	H263C156O92N20	3888.651	3.4009	1944.8228	2	99.06	1	24.63	9728	96.58	74.99
56.04	12618	1.51	12613	VTSPNITVTLK_6_5_1_3	H280C167O100N21	4179.7464	0.0299	1045.6925	4	99.65	1	66.41	12622	95.07	86.47
59.03	13935	1.55	13927	VTSPNITVTLK_6_5_1_3	H280C167O100N21	4179.7464	0.0837	1393.9208	3	99.38	1	34.57	13938	95.13	76.96
56.82	12985	1.55	12981	VTSPNITVTLK_6_5_1_3	H280C167O100N21	4179.7464	0.5144	1393.9214	3	99.76	1	29.72	12988	97.77	77.36
56.21	12701	1.56	12684	VTSPNITVTLK_6_5_1_3	H280C167O100N21	4179.7464	0.634	1393.9198	3	99.57	1	70.95	12704	97.62	89.62

56.24	12714	1.54	12708	VTSPNITVTLK_6_5_1_3	H280C167O100N21	4179.7464	0.634	1393.9198	3	99.57	1	75.07	12716	97.99	91.11
57.53	13297	1.5	13290	VTSPNITVTLK_6_5_1_3	H280C167O100N21	4179.7464	0.8912	1045.6934	4	99.23	1	45.05	13300	92.84	78.5
55.49	12371	1.54	12367	VTSPNITVTLK_6_5_1_3	H280C167O100N21	4179.7464	0.9211	1393.9194	3	99.48	1	67.18	12375	97.88	88.67
57.63	13335	1.55	13328	VTSPNITVTLK_6_5_1_3	H280C167O100N21	4179.7464	1.0886	1393.9222	3	99.62	1	16.89	13338	93.64	70.61
55.21	12244	1.53	12236	VTSPNITVTLK_6_5_1_3	H280C167O100N21	4179.7464	1.274	1045.6938	4	99.76	1	29.64	12248	92.17	73.41
55.84	12522	1.54	12518	VTSPNITVTLK_6_5_1_3	H280C167O100N21	4179.7464	1.274	1045.6938	4	99.76	1	22.85	12526	91.99	71.25
55.72	12472	1.62	12453	VTSPNITVTLK_6_5_1_3	H280C167O100N21	4179.7464	1.3338	2090.3799	2	99.1	1	46.96	12475	89.11	76.46
61.71	15195	1.59	15185	VTSPNITVTLK_6_5_1_3	H280C167O100N21	4179.7464	1.3757	1393.9226	3	99.54	1	48.55	15198	95.54	81.44
54.91	12100	1.58	12082	VTSPNITVTLK_6_5_1_3	H280C167O100N21	4179.7464	1.4475	1393.9227	3	99.01	1	34.53	12103	93.96	76.13
54.94	12114	1.57	12105	VTSPNITVTLK_6_5_1_3	H280C167O100N21	4179.7464	1.4475	1393.9227	3	99.01	1	40.64	12117	94.13	78.08
56.57	12869	1.61	12858	VTSPNITVTLK_6_5_1_3	H280C167O100N21	4179.7464	1.8123	2090.3809	2	99.57	1	77.07	12872	92.26	87.7
58.31	13616	1.54	13609	VTSPNITVTLK_6_5_1_3	H280C167O100N21	4179.7464	3.2418	1393.9252	3	99.51	1	28.05	13619	95.36	75.17
50.84	9944	1.52	9934	VTSPNITVTLK_6_6_0_2	H266C158O93N21	3945.6725	0.8744	1315.8949	3	99.67	1	32.99	9948	93.11	75.07
51.02	10030	1.5	10023	VTSPNITVTLK_6_6_0_2	H266C158O93N21	3945.6725	0.8744	1315.8949	3	99.67	1	37.38	10034	90.83	74.8
47.98	8612	1.37	8601	VTSPNITVTLK_7_6_1_1	H269C159O94N20	3962.6878	0.6687	1321.5687	3	99.96	1	69.11	8615	96.26	88.11
55.71	12466	1.6	12453	VTSPNITVTLK_7_6_1_3	H303C181O110N22	4544.8786	0.1595	1136.9757	4	99.43	1	65.4	12470	98.04	88.25
55.75	12482	1.61	12481	VTSPNITVTLK_7_6_1_3	H303C181O110N22	4544.8786	0.1595	1136.9757	4	99.43	1	66.7	12485	98.54	88.99
54.46	11861	1.6	11854	VTSPNITVTLK_7_6_1_3	H303C181O110N22	4544.8786	0.5116	1136.9761	4	99.87	1	47.21	11864	98.24	82.93
55.08	12180	1.58	12171	VTSPNITVTLK_7_6_1_3	H303C181O110N22	4544.8786	0.5116	1136.9761	4	99.87	1	67.21	12184	94.12	86.05
56.49	12831	1.6	12809	VTSPNITVTLK_7_6_1_3	H303C181O110N22	4544.8786	0.7756	1136.9764	4	99.44	1	25.34	12833	91.63	71.75
58.27	13601	1.6	13588	VTSPNITVTLK_7_6_1_3	H303C181O110N22	4544.8786	1.5072	1515.6337	3	98.3	1	86.1	13605	51.48	61.86
57.96	13478	1.62	13469	VTSPNITVTLK_7_6_1_3	H303C181O110N22	4544.8786	1.5677	1136.9773	4	99.34	1	34.23	13481	97.37	78.43
55.8	12506	1.59	12498	VTSPNITVTLK_7_6_1_3	H303C181O110N22	4544.8786	1.9032	1515.6343	3	98.32	1	79.45	12509	100	93.84
72.46	20428	1.51	20414	VTSPNITVTLK_7_6_1_4	H320C192O118N23	4835.974	0.61	1612.6642	3	99.42	1	18.65	20431	93.54	71.07
71.57	19997	1.48	19981	VTSPNITVTLK_7_6_1_4	H320C192O118N23	4835.974	1.1063	1612.665	3	98.49	1	76.37	19998	72.17	73.43
54.41	11837	1.59	11831	VTSPNITVTLK_8_7_1_3	H326C195O120N23	4910.0108	0.8859	1637.3436	3	99.53	1	47.93	11840	84.24	73.35
68.35	18438	1.57	18429	VTSPNITVTLK_8_7_1_4	H343C206O128N24	5201.1062	0.0625	1301.0325	4	99.54	1	21.75	18441	98.95	75.79
68.48	18504	1.6	18495	VTSPNITVTLK_8_7_1_4	H343C206O128N24	5201.1062	0.0625	1301.0325	4	99.54	1	27.48	18507	89.46	70.87
69.47	18997	1.63	18990	VTSPNITVTLK_8_7_1_4	H343C206O128N24	5201.1062	1.5526	1301.0304	4	98.24	1	26.59	19002	88.26	69.76
64.94	16697	1.69	16683	VTSPNITVTLK_9_8_1_4	H366C220O138N25	5566.2384	0.6962	1392.3145	4	99.47	1	9.21	16700	94.25	68.74
63.56	16048	1.62	16029	VTSPNITVTLK_9_8_1_4	H366C220O138N25	5566.2384	1.3429	1392.3136	4	98.92	1	7.08	16049	87.49	63.37
